# Supplementary material for: Knowledge, attitudes, and practices toward the novel coronavirus among Bangladeshis: Implications for mitigation measures
Source: PLoS One. 2020 Sep 2;15(9):e0238492. doi: 10.1371/journal.pone.0238492 (PMC7467312; doi:10.1371/journal.pone.0238492)
Supplement: S1 Table — (DOC) [file pone.0238492.s001.doc]

**S1 Table. KAP questionnaire for the COVID-19 for online survey in Bangladesh.**

| 1. **Respondents’ Profile** | | |
| --- | --- | --- |
| **Questions** | **Responses** | **References** |
| What is your gender? | - Woman - Man |  |
| What is your age group (years)? | - 18-25 years - 26-35 years - 36-45 years - 46-65 years - Over 65 years |  |
| What is your highest education attainment? | - Primary - Secondary - Diploma - University. |  |
| What is your occupation? | - Unemployed - Student - Retired - Professionals (teachers, physicians, bankers, NGO professionals, Corporate professionals, etc.) - Government staff - Homemakers |  |
| Where is your current residence location? | Please mention name of district, upazila, city/town, village |  |
| 1. **Knowledge** | | |
| How do you perceive COVID-19? | - A deadly disease with certainty of death, - A deadly disease, curable and low mortality rate^*^, - A rumour which is being spread through public or media, - A curse from the God | [38] |
| COVID-19 was emerged due to following reasons. Multiple answer is allowed. | - Food habit (e.g. eating or contacting wild animals), - Severe destruction of natural environment, - A disease in temperate (cold weather) countries, Natural justice, None of above* | [39] |
| The main clinical symptoms of COVID-19 are fever, fatigue, dry cough, and breathing difficulty. | - Yes^*^, - No, - Maybe | [38] |
| Currently there is no effective cure for COVID-2019, but early symptomatic and supportive treatment can help most patients recover from the infection | - Yes^*^, - No, - Maybe | [38 39] |
| Only elderly people having chronic illnesses and other health complications are more likely to be seriously affected | - Yes*, - No, - Maybe | [38] |
| Persons with COVID-2019 having no fever cannot infect to others. | - Yes, - No*, - Maybe | [39] |
| The COVID-19 spreads via respiratory droplets (from coughing, sneezing) of infected people. | - Yes* - No - Maybe | [39] |
| Ordinary residents can wear general medical masks to prevent COVID-19 infection. | - Yes - No* - Maybe | [39] |
| It is not necessary for children and young adults to take measures to prevent the infection by the COVID-19. | - Yes - No* - Maybe | [38] |
| What do you understand by quarantine? | - Stay at home with family members - Stay at a separate room, and no contact with family members* - Stay at home and can go outside - No clear understanding | [40] |
| In general, what is the quarantine period? | - 1 week - 2 weeks* - A few days - Do not know | [40] |
| When should we go for quarantine? Multiple answer is allowed. | - When feel having COVID-19 symptoms - When get contact with infected people* - When people return from visiting infected countries/areas* - Don’t know | [40] |
| Isolation and treatment of COVID-19 infected people are effective ways to reduce the spread of the virus. | - Yes* - No - Maybe | [41] |
| Comparing with other affected nations, what is the possibility of COVID-19 spread in Bangladesh? Multiple answer is allowed. | - No possibility of spread - Least possibility of spread - Moderate possibility of spread - High possibility of spread* - Very high possibility of spread | [42] |
| What could be the possible reasons of COVID-19 spread in Bangladesh if it happens? Multiple answer is allowed. | - Lack of awareness among people* - High density of population* - Return of immigrants from affected countries* - No proper quarantine* - Lack of advancement in the medical facilities* - Lack of enough preventive measures taken by the government* | [42,43] |
| What should be the priority actions for government to control the spread of COVID-19? Multiple answer is allowed. | - Strict prohibition of public gatherings (e.g. campaign, religious & social events, etc.) for next three months* - Identify and quarantine all people returned from infected countries* - Develop enough appropriate quarantine facilities across the country* - Complete lockdown of infected areas until further notice* - Impose strict movement control of general people for at least few weeks* - Mass awareness creation on preventive measures* - Encourage working from home* - Encourage digital learning for students* - Conduct all meetings, seminars and conferences online* - Develop medical facilities across the country* | [42-45] |
| **C. Attitudes** | | |
| Do you like to stay at home for certain period (14 days) to prevent COVID-19 spread if government will order so? | - Yes* - No - Not possible due to work | [24 29] |
| Do you think that social distancing (e.g. stay 1-2 m apart, avoid crowds, etc.) can prevent COVID-19 spread? | - Yes* - No - Maybe | [40] |
| Do you agree that we should cancel business/recreational trips at this time? | - Yes* - No - Maybe | [40] |
| Do you believe that working from home can help control COVID-19? | - Yes* - No - Maybe | [40] |
| Do you agree that government has taken sufficient preventive measures to prevent the spread of COVID-19? | - Yes - No* - Not enough* | [43] |
| Do you agree that government should take preventive measures when COVID-19 was first reported in China? | - Yes* - No - Maybe | [24] |
| Do you think COVID-19 can cause massive fatality in Bangladesh? | - Yes - No - Maybe* | [46,47] |
| Do you believe that COVID-19 will not be epidemic in Bangladesh due to following reasons? Multiple answer allowed. | - Relatively warm and humid weather - Immune system of Bangladeshi people is high - People of Bangladesh believe in God - People of Bangladesh are kind to each other - People of Bangladesh has experience to face disaster - None of above* | [44] |
| Do you agree that our health support providers (e.g. doctors, nurses, support staff) are under serious threat when they treat infected people? | - Yes* - No - Maybe | [48] |
| Do you think that government has ensured enough protective measures for health support providers? | - Yes - No* - Maybe | [46] |
| 1. **Practices** | | |
| Do you go to crowded areas now-a-days? | - Yes - No* - Sometimes | [49] |
| Do you allow your children/youngers to go for outdoor activities? | - Yes - No* - Sometimes | [49] |
| Do you and family members use mask when go outside? | - Yes - No* - Sometimes | [49] |
| Have you started working from home in last few weeks due to outbreak of COVID-19? | - Yes* - No - Sometimes | [46] |
| How would you rate the awareness level of the people living around you regarding COVID-19? | - No precautionary measures at all - Little awareness has grown so far* - Some precautionary measures have been taken - Awareness level is on rise - People around me are highly aware and careful | [24] |
| How would you rate the medical facilities in Bangladesh to handle COVID-19? | - Very poor facilities are available so far* - Health facilities are available for limited number of people - Gradual advancement in health care is noticeable to deal with COVID-19 - The country has quite a good facility to prevent COVID-19 - Medical facilities are highly appreciable, and it can prevent the spread of COVID-19 | [29] |
| Are the people in your area/district already panic about COVID-19? | - Yes* - No - Maybe | [29] |
| Are you feeling anxious/stressed/depressed/helpless thinking about the outbreak of COVID-19? | - Yes* - No - Maybe | [29,44] |
| If above answer is YES, then please rate your level of feelings. | - Little - Moderate - High* - Extreme | [44] |

Note: * indicates right answers
