# Supplementary material for: Knowledge, attitudes, and practices toward the novel coronavirus among Bangladeshis: Implications for mitigation measures
Source: PLoS One. 2020 Sep 2;15(9):e0238492. doi: 10.1371/journal.pone.0238492 (PMC7467312; doi:10.1371/journal.pone.0238492)
Supplement: S2 Table — (DOCX) [file pone.0238492.s002.docx]

**S2 Table. Cut point of knowledge, attitudes and practices.**

| **Variable** | **Frequency** | **Percentage** | **Cumulative Percentage** |
| --- | --- | --- | --- |
| **Knowledge score** | | |  |
| 2.5 | 1 | 0.1 | 0.1 |
| 4 | 3 | 0.2 | 0.3 |
| 4.5 | 1 | 0.1 | 0.4 |
| 5 | 5 | 0.3 | 0.7 |
| 5.5 | 3 | 0.2 | 0.9 |
| 6 | 18 | 1.1 | 2.0 |
| 6.5 | 18 | 1.1 | 3.1 |
| 7 | 49 | 3.1 | 6.2 |
| 7.5 | 65 | 4.1 | 10.3 |
| 8 | 109 | 6.9 | 17.2 |
| 8.5 | 123 | 7.7 | 24.9 |
| 9 | 234 | 14.7 | 39.6 |
| 9.5 | 170 | 10.7 | 50.3 |
| 10 | 266 | 16.7 | 67.0 |
| 10.5 | 145 | 9.1 | 76.1 |
| 11 | 211 | 13.3 | 89.4 |
| 11.5 | 73 | 4.6 | 94.0 |
| 12 | 74 | 4.7 | 98.7 |
| 12.5 | 12 | 0.8 | 99.4 |
| 13 | 9 | 0.6 | 100.0 |
| Total | 1589 | 100.0 |  |
| Poor knowledge (score: 1.0-10.4) | 1065 | 67.0 | 67.0 |
| Good knowledge (score:>10.4) | 524 | 33.0 | 100.0 |
| **Attitude score** | | |  |
| 4 | 3 | 0.2 | 0.2 |
| 4.5 | 12 | 0.8 | 1.0 |
| 5 | 15 | 0.9 | 1.9 |
| 5.5 | 19 | 1.2 | 3.1 |
| 6 | 56 | 3.5 | 6.6 |
| 6.5 | 55 | 3.5 | 10.1 |
| 7 | 158 | 9.9 | 20.0 |
| 7.5 | 109 | 6.9 | 26.9 |
| 8 | 329 | 20.7 | 47.6 |
| 8.5 | 186 | 11.7 | 59.3 |
| 9 | 564 | 35.5 | 94.8 |
| 9.5 | 29 | 1.8 | 96.6 |
| 10 | 54 | 3.4 | 100.0 |
| Total | 1589 | 100.0 |  |
| Poor attitude (score: 1.0-8.0) | 756 | 47.6 | 47.6 |
| Good attitude (score:>8.0) | 833 | 52.4 | 100.0 |
| **Practice score** | | |  |
| 1 | 1 | 0.1 | 0.1 |
| 1.5 | 1 | 0.1 | 0.1 |
| 2 | 6 | 0.4 | 0.5 |
| 2.5 | 3 | 0.2 | 0.7 |
| 3 | 27 | 1.7 | 2.4 |
| 3.5 | 11 | 0.7 | 3.1 |
| 4 | 81 | 5.1 | 8.2 |
| 4.5 | 17 | 1.1 | 9.3 |
| 5 | 260 | 16.4 | 25.6 |
| 5.5 | 39 | 2.5 | 28.1 |
| 6 | 432 | 27.2 | 55.3 |
| 6.5 | 33 | 2.1 | 57.3 |
| 7 | 439 | 27.6 | 85.0 |
| 7.5 | 13 | 0.8 | 85.8 |
| 8 | 226 | 14.2 | 100.0 |
| Total | 1589 | 100.0 |  |
| Poor practice (score: 1.0-6.4) | 878 | 55.3 | 55.3 |
| Good practice (score:>6.4) | 711 | 44.7 | 100.0 |
