# Supplementary material for: Knowledge, attitudes, and practices toward the novel coronavirus among Bangladeshis: Implications for mitigation measures
Source: PLoS One. 2020 Sep 2;15(9):e0238492. doi: 10.1371/journal.pone.0238492 (PMC7467312; doi:10.1371/journal.pone.0238492)
Supplement: S3 Table — (DOC) [file pone.0238492.s003.doc]

**S3 Table. Categories of responses to attitudes and practices used for logistic regression.**

| **Variable** | | | |  | **Logistic** | |
| --- | --- | --- | --- | --- | --- | --- |
| **Attitudes** | | | | | | |
| Social distancing | | | |  |  | |
| Yes | No | May be |  |  | Yes | Others |
|  |  |  |  |  |  |  |
| Cancel business / recreational trips | | |  |  |  |  |
| Yes | No | May be |  |  | Yes | Others |
| Working from home | | |  |  |  |  |
| Yes | No | May be |  |  | Yes | Others |
| Sufficient preventive measures by Government | | |  |  |  |  |
| Yes | No | Not enough |  |  | No & Not enough | Other |
| Response from Govt. after reports from Wuhan | | |  |  |  |  |
| Yes | No | May be |  |  | Yes | Others |
| Massive fatality or not | | |  |  |  |  |
| Yes | No | May be |  |  | May be | Others |
| Seriousness of threat to healthcare providers | | |  |  |  |  |
| Yes | No | May be |  |  | Yes | Others |
| Protection for healthcare providers | | |  |  |  |  |
| Yes | No | May be |  |  | No | Others |
| **Practices** | | | | | | |
| Visit to crowded areas now-a-days | | | | | | |
| Every day | No | Sometimes | Yes |  | No | Other |
| Allowing children outdoor activities |  |  |  |  |  |  |
| No | Sometimes | Yes |  |  | No | Other |
| Using face mask while going outside |  |  |  |  |  |  |
| No | Sometimes | Yes |  |  | Yes | Other |
| Level of awareness among the neighbors |  |  |  |  |  |  |
| Awareness level is on rise | Little awareness has grown so far | No precautionary measures undertaken at all | People around me are highly aware and careful | Some precautionary measures have been taken | Awareness level is on rise & Little awareness has grown so far | Other |
| Rating the medical facilities |  |  |  |  |  |  |
| Gradual advancement in health care is noticeable to deal with covid-19 | Health facilities are available for limited number of people | Medical facilities are highly appreciable, and it can prevent the spread of covid-19 | The country has quite a good facility to prevent covid-19 | Very poor facilities are available so far | Very poor facilities are available so far | Other |
| Panic among neighbors |  |  |  |  |  |  |
| Maybe | No | Yes |  |  | Yes | Other |
| Anxious/ stressed/ due to COVID |  |  |  |  |  |  |
| Maybe | No | Yes |  |  | Yes | Other |
| Level of stress /anxiety due to COVID |  |  |  |  |  |  |
| Extreme | High | Little | Moderate |  | High | Other |
