# Supplementary material for: Knowledge, attitudes, and practices toward the novel coronavirus among Bangladeshis: Implications for mitigation measures
Source: PLoS One. 2020 Sep 2;15(9):e0238492. doi: 10.1371/journal.pone.0238492 (PMC7467312; doi:10.1371/journal.pone.0238492)
Supplement: S4 Table — (DOCX) [file pone.0238492.s004.docx]

**S4 Table. Pearson’s correlation coefficients for knowledge, attitudes, and practices.**

| **Correlations** | | | | |
| --- | --- | --- | --- | --- |
|  |  | Knowledge | Attitudes | Practices |
| Knowledge | Pearson Correlation | 1 | 0.249^**^ | 0.033 |
|  | Sig. (2-tailed) |  | 0.001 | 0.188 |
|  | N | 1589 | 1589 | 1589 |
| Attitudes | Pearson Correlation | 0.249^**^ | 1 | 0.148^**^ |
|  | Sig. (2-tailed) | 0.001 |  | 0.001 |
|  | N | 1589 | 1589 | 1589 |
| Practices | Pearson Correlation | 0.033 | 0.148^**^ | 1 |
|  | Sig. (2-tailed) | 0.188 | 0.001 |  |
|  | N | 1589 | 1589 | 1589 |
| **. Correlation is significant at ≤0.01 level (2-tailed). | | | |  |
