# Supplementary material for: Knowledge, attitudes, and practices toward the novel coronavirus among Bangladeshis: Implications for mitigation measures
Source: PLoS One. 2020 Sep 2;15(9):e0238492. doi: 10.1371/journal.pone.0238492 (PMC7467312; doi:10.1371/journal.pone.0238492)
Supplement: Attachment — Submitted filename: Response to Reviewers.docx [file pone.0238492.s006.docx]

**Response to Reviewers**

PONE-D-20-12138
Knowledge, attitude and practice towards the novel corona virus among Bangladeshi people: Implications for mitigation measures

We thank the academic editor and the reviewers for their feedback. We found all the reviewers’ comments to be usefully constructive, and we appreciate the time taken to provide such thoughtful and thorough feedback. We would also like to thank the reviewers for their positive comments. Both reviewers, however, made some suggestions around ways to improve the manuscript which we found useful and have addressed.

In the following sections, we provide more detail on these changes, addressing the reviewers’ comments one by one.

**Editor’s Comments**

Comments 1: A rebuttal letter that responds to each point raised by the academic editor and reviewer(s). You should upload this letter as a separate file labeled 'Response to Reviewers'.

Response: We addressed all comments raised by the editor and reviewers and uploaded a file labeled “Response to Reviewers”.

Comments 2: A marked-up copy of your manuscript that highlights changes made to the original version. You should upload this as a separate file labeled 'Revised Manuscript with Track Changes'.

Response: We have done so accordingly.

Comments 3: An unmarked version of your revised paper without tracked changes. You should upload this as a separate file labeled 'Manuscript'.

Response: We have done so accordingly.

Comments 4: If you would like to make changes to your financial disclosure, please include your updated statement in your cover letter.

Response: We have not made any changes to financial disclosure.

Comments 5: Guidelines for resubmitting your figure files are available below the reviewer comments at the end of this letter.

Response: We followed the guidelines

Comments 6: If applicable, we recommend that you deposit your laboratory protocols in protocols.io to enhance the reproducibility of your results. Protocols.io assigns your protocol its own identifier (DOI) so that it can be cited independently in the future. For instructions see: <http://journals.plos.org/plosone/s/submission-guidelines#loc-laboratory-protocols>

Response: Our study does not have any laboratory protocol to deposit.

Comments 7: When submitting your revision, please ensure that your manuscript meets PLOS ONE's style requirements, including those for file naming. The PLOS ONE style templates can be found at

https://journals.plos.org/plosone/s/file?id=wjVg/PLOSOne_formatting_sample_main_body.pdf and

https://journals.plos.org/plosone/s/file?id=ba62/PLOSOne_formatting_sample_title_authors_affiliations.pdf

 Response: We followed PLOS ONE style in this revision.

Comments 8: Thank you for stating the following in the Funding Section of your manuscript:

"The study has been financed by the team of researchers involved in the work, and no external funding was available."

We note that you have provided funding information that is not currently declared in your Funding Statement. However, funding information should not appear in the Acknowledgments section or other areas of your manuscript. We will only publish funding information present in the Funding Statement section of the online submission form.

Please remove any funding-related text from the manuscript and let us know how you would like to update your Funding Statement. Currently, your Funding Statement reads as follows:

"The author(s) received no specific funding for this work"

 Response: We removed funding statement from the revised manuscript

Comments 9: We note that Figure 'S1 Fig' in your submission contain map images which may be copyrighted. All PLOS content is published under the Creative Commons Attribution License (CC BY 4.0), which means that the manuscript, images, and Supporting Information files will be freely available online, and any third party is permitted to access, download, copy, distribute, and use these materials in any way, even commercially, with proper attribution. For these reasons, we cannot publish previously copyrighted maps or satellite images created using proprietary data, such as Google software (Google Maps, Street View, and Earth). For more information, see our copyright guidelines: http://journals.plos.org/plosone/s/licenses-and-copyright.

We require you to either (1) present written permission from the copyright holder to publish these figures specifically under the CC BY 4.0 license, or (2) remove the figures from your submission:

Response: We removed “Figure S1” from revised manuscript

**Reviewers' comments**:


**Reviewer #1**:

Comments 1: The authors should go throughout the manuscript and carefully scrutinize many grammatical errors and punctuations. The authors should ask a native speaker of English to improve the manuscript and abstract.

Response: This revised paper was edited by Elsevier English Editing Service (a certificate is attached)

Comments 2: This manuscript reports knowledge, attitude and practice toward COVID-19 among general population of Bangladesh. Under COVID-19 outbreak all over the world, the topic is really important. However, design of this study and structure have some issues and limitations. The authors should separate the results from the discussion section for better understanding of the manuscript.

Response: We understand that submitted manuscript has some issues to be improved. We tried our best to improve this paper based on reviewers’ comments, added a section on Limitations of this Study. We also separated Results and Discussion sections.

Comments 3: Although COVID-19 outbreak is a recent global issue, the authors should support the discussion of their study by recent articles regarding KAP towards COVID-19 in other areas. articles concerning KAP towards COVID-19 have been recently published both in neighboring countries and other parts of the world. The authors should revise their discussion section and add more comparison to similar studies in other areas. (e.g: Erfani A, Knowledge, Attitude and Practice toward the Novel Coronavirus (COVID-19) Outbreak: A Population-Based Survey in Iran.2020 / Zhong B-L et al Knowledge, attitudes, and practices towards COVID-19 among Chinese residents during the rapid rise period of the COVID-19 outbreak: a quick online cross-sectional survey. Int J Biol Sci. 2020 / Srichan P, et al. Knowledge, Attitude and Preparedness to Respond to the 2019 Novel Coronavirus (COVID-19) Among the Bordered Population of Northern Thailand in the Early Period of the Outbreak: A Cross-Sectional Study. 2020).

Response: Thanks a lot for helping us with important references. We discussed our results with above and additional references.

Comment 4: The authors should revise the conclusion to a shorter and clearer version. as much of the information in this section are more appropriate for the discussion section.

Response: We revised Conclusions in a shorter form.

Comments 5: The authors should add a section regarding their limitation in their study, e.g. the selection bios which particularly consists of higher educated individuals, etc.

Response: We have added a section on Limitations of this Study where we described all of our shortcomings of the study.

Comments 6: One of the big drawbacks of this study is the method of sample collection. The authors used a web-based survey; however, the details of recruitment were unclear. To call participants, the authors seemed to provide the questionnaire via email and Facebook, but if so, who saw the link? What is the response rate? In other words, what is the “mother population” of this survey? This study had a serious selection bias problem along with small sample size. The aim of this study was to understand the status of knowledge regarding COVID-19. The sampling method in this study is not suitable for this purpose under the unclear mother population.

Response: We do agree that sampling was one of the limitations of this study. Mother population was unknown. The survey link was circulated to Facebook friends and emailed to people known to authors of this paper. As population was unknown, it was not possible to estimate the response rate. We addressed this in Methods section.

Comments 7: The authors allocated the same scoring for incorrect and “Maybe”. What is the reason and validity of these allocations? Since the Likert scale scoring system differentiates “don’t know” from “incorrect” answer. In other words, does incorrect knowledge and no knowledge have the same impact on disease control and transmission among the population? If the authors allocated different scoring for “incorrect answers” and “don’t know” would there be any difference in results?

Response: In this revision, we changed the scoring approach. We used score 1 for each correct answer, 0.5 for maybe answer and 0 for each incorrect answer. With this new scoring approach we revised Table 2.

Comments 8: The authors failed to provide a framework for the analyzing section of the data. The work requires an extremely detailed use of language throughout the paper to ensure that shared meaning is held among the readers and the authors. The authors tended to rely on vague terms and need more stringent attention to detail in language to better support the authors’ suppositions.

Response: Our apology. We do not understand the comment “The authors failed to provide a framework for the analyzing section of the data”. However, we elaborated Methods and Data Analysis. As stated above, the revised paper was checked by a professional English editor.

Comments 9: The authors need to explain in the method section how the sample size was determined prior to data gathering. In other words, what was the estimated sample size?

Response: As our “mother population” was unknown, we could not determine sample size prior to data gathering. In fact, we stopped receiving responses when there were very low survey participation.

Comments 10: The authors mention in their ethical statement that the participants provided informed consents. Please clarify how these consents where obtained (oral or written).

Response: We stated following statement to clarify this: “Before participating in the survey, respondents had to answer a Yes/No question to confirm their consent to participate voluntarily. After conformation of this question, the respondents were directed to the complete the questionnaire”.

**Reviewer #2**

Comments 1: This paper addressed the KAP about COVID-19 among Bangladeshi people. The authors conducted a cross-sectional study and received complete response from 1589 participants. Considering socioeconomic and literacy in Bangladesh, the findings of this study is very significant. However, I have some major concerns.

Response: Thanks for your kind notes.

Comments 2: This study may not be representative of Bangladeshi population as only 4.2% of the participants were recruited from the cohort with education level secondary or below and 95% from university graduate. However, the prevalence of university graduate in the country is may be less than 10%. Thus the findings of this study may not represent Bangladeshi population, instead it may represent Bangladeshi people who have completed university degree.

Response: We completely agree with this comment. As the respondents were authors’ Facebook friends and friends of friends’, they were mostly educated. This was one of the limitations of this study, and researcher addressed this as one of the limitations of online study.

Comments 3: Since, English is not a mother language, the English version of the questionnaire further increases selection/participation bias in the study.

Response: This was true, and one of our study limitations.

Comments 4: I would recommend to report the actual distribution of education as well as occupation level in Bangladesh,

Response: From Bangladesh Statistical data, we have current distribution of education and occupation level in Bangladesh as follow:

Education: Primary (67.89%), Secondary (30.95%) and post-secondary (1.16%). In case of occupation, the distribution is Agriculture (38.58%), Industry (21.26%) and Service sector (40.16%) (Source: Bangladesh Bureau of Statistics, 2017)

Comments 5: The reported data in line 253 do not match with the data provided in the Table 2.

Response: We have corrected this.

Comments 6: The multiple logistic regression analysis results in Table 7A and 7B are confusing. Clearly, the analysis has not been done for attitude or practice score; instead it has been done for various component of attitude and practice. This should be reflected from the title of the table. Some of the outcome variables in these table have more than two categories. I am wondering how the logistic regression analysis was performed for these outcomes.

Response: Absolutely right. The analysis was not done for attitudes/practices’ scores, but for various attributes of attitudes and practices. We have revised the Table caption to avoid the confusion.
For Logistic regression, variable were categorized into two categories. All “Yes” responses as YES, and all “No and Maybe” responses as OTHERS for a number of questions. These YES and OTHERS categories were used for regression analysis.

Comments 7: In the above table, I notice some reported ORs are exceptionally very high (e.g., Table 7A, OR related to Age>65yrs is 3.211E7). How do you interpret this OR. This arises because there was not enough participants in this age group. The authors may combine this age group with the preceding group.

Response: This is absolutely valid comment. In this revision, we have combined the suggested age groups.

Comments 8: I would recommend to report 95% CI for reported OR in Table 7A & B.

Response: We have provided 95% CI in Table 7A & 7B.

Comments 9: I am concern that the authors choose to present results and discussion in the same section.

Response: We have separated Results and Discussion

Comments 10: I find the conclusion section very long. I would recommend to summarise the findings in a short section.

Response: We have shorten Conclusion

Comments 11: What are the limitation of this study; this need to be stated.

Response: We have added a section on Limitations of this Study
