# Supplementary material for: Knowledge, attitudes, and practices toward the novel coronavirus among Bangladeshis: Implications for mitigation measures
Source: PLoS One. 2020 Sep 2;15(9):e0238492. doi: 10.1371/journal.pone.0238492 (PMC7467312; doi:10.1371/journal.pone.0238492)
Supplement: Attachment — Submitted filename: Response to Reviewers.docx [file pone.0238492.s007.docx]

**Response to Reviewers**

PONE-D-20-12138
Knowledge, attitudes and practices toward the novel coronavirus among Bangladeshis: Implications for mitigation measures

We thank the academic editor and the reviewers for their further feedback. We found all the reviewers’ comments to be usefully constructive, and we appreciate the time taken to provide such thoughtful and thorough feedback. Reviewers made some more suggestions to improve the manuscript which we found useful and have addressed.

In the following sections, we provide more detail on these changes, addressing the reviewers’ comments one by one.

**Editor’s Comments**

Comments: Thank you for submitting your manuscript to PLOS ONE. After careful consideration, we feel that it has merit but does not fully meet PLOS ONE’s publication criteria as it currently stands. Therefore, we invite you to submit a revised version of the manuscript that addresses the points raised during the review process.

Response: Thanks for considering our revised paper for further revision. We have addressed all comments of reviewers.

Comments: A rebuttal letter that responds to each point raised by the academic editor and reviewer(s). You should upload this letter as a separate file labeled 'Response to Reviewers'.

Response: We have addressed all comments and uploaded a file named “Response to Reviewers”.

Comments: A marked-up copy of your manuscript that highlights changes made to the original version. You should upload this as a separate file labeled 'Revised Manuscript with Track Changes'.

Response: We have uploaded a marked-up copy named “Revised Manuscript with Track Changes”.

Comments: An unmarked version of your revised paper without tracked changes. You should upload this as a separate file labeled 'Manuscript'.

Response: We have uploaded revised paper named “Manuscript”.

**Reviewers' comments**

Reviewer #1:

Comments: The authors have done a great job in addressing the concerns and comments and have significantly improved the manuscript. However, a few concerns still remain which can help improve the manuscript before considering it for publication.

Response: Thanks for your kind comments. We have addressed all concerns in this revision (R2).

Comments: In “table 2” and “supplementary table 4” please report <0.001 instead of 0.000.

Response: We have corrected accordingly.

Comments: I recommend adjusting the design of the tables, particularly the borders, since there is no consistency and seems that the borders are inserted randomly. An option can be using three-line tables.

Response: We re-designed all Tables following your comments.

Comments: In table 7 what do the numbers represent? They seem to be OR and 95%CI, if so, please state in the table.

Response: The numbers represent odds ratio (OR) followed by 95% CI in parenthesis.

Comments: The conclusion section of the manuscript still needs improvement and can be shortened and improved, as it should demonstrate the final result and what the authors have achieved and how it can be used or implanted.

Response: We have tried to shortened conclusion.

Comments: I recommend reporting the “cumulative percentage” in the first table of the “supplementary 2” file as it is more valuable to the readers. The authors could even express the table as a cumulative frequency/percentage graph if they choose.

Response: We provided cumulative percentage in Table S2.
