# Supplementary material for: Knowledge, attitudes, and practices toward the novel coronavirus among Bangladeshis: Implications for mitigation measures
Source: PLoS One. 2020 Sep 2;15(9):e0238492. doi: 10.1371/journal.pone.0238492 (PMC7467312; doi:10.1371/journal.pone.0238492)
Supplement: Attachment — Submitted filename: Response to Reviewers.docx [file pone.0238492.s008.docx]

**Response to Reviewers**

PONE-D-20-12138R2
Knowledge, attitudes and practices toward the novel coronavirus among Bangladeshis: Implications for mitigation measures

We thank the academic editor for further feedback. We found all comments to be usefully constructive, and we appreciate the time taken to provide such thoughtful and thorough feedback. We have addressed all suggestions to improve the manuscript.

In the following sections, we provide more detail on these changes, addressing the comments one by one.

**Editor’s Comments**

Comments: Thank you for submitting your manuscript to PLOS ONE. After careful consideration, we feel that it has merit but does not fully meet PLOS ONE’s publication criteria as it currently stands. Therefore, we invite you to submit a revised version of the manuscript that addresses the points raised during the review process.

Response: Thanks for considering our revised paper for further revision. We have addressed all comments in this revision.

Comments: A rebuttal letter that responds to each point raised by the academic editor and reviewer(s). You should upload this letter as a separate file labeled 'Response to Reviewers'.

Response: We have addressed all comments and uploaded a file named “Response to Reviewers”.

Comments: A marked-up copy of your manuscript that highlights changes made to the original version. You should upload this as a separate file labeled 'Revised Manuscript with Track Changes'.

Response: We have uploaded a marked-up copy named “Revised Manuscript with Track Changes”.

Comments: An unmarked version of your revised paper without tracked changes. You should upload this as a separate file labeled 'Manuscript'.

Response: We have uploaded revised paper named “Manuscript”.

Comments: Table 2: footnote- should be *** Significant at 0.01 level; Remove Less than or equal to. Also change in other tables 7A and 7B also.

Response: We have changed accordingly in Table 2, Table 7, and Table 8

Comments: Pages 17-21: 95%CI is missing in the interpretation of results for all covariates. CI should be included. For example, Page 17, 3rd line of the last paragraph: "working from home (OR 1.708, 95% CI, p<0.01)"- This should be written as "working from home (OR=1.708, 95%CI:1.198-2.437, p<0.01). Include CI in all places. Follow the PLOS ONE guidelines or refer previously published articles in PLOS ONE.

Response: We have added values of 95% Confidence Interval in results following a recent PLOS ONE paper.

Comments: Table 7A and 7B: Include in the Table Title at the end within a bracket- Values are Adjusted Odds Ratios followed by 95% Confidence Interval in parenthesis, instead of in the footnote.

Response: We have split Table 7A and 7B into Table 7 and Table 8, and moved ‘values are adjusted odds ratios followed by 95% Confidence Interval in parenthesis at the end of Table title.

Comments: Table 7A and 7B: For all reference category, include OR is 1.00 (NO CI) instead of leaving it blank.

Response: We provided OR 1.00 accordingly.

Comments: Table 7B and 7B still needs proper formatting. All are mixed. For example, Table 7A: Gender and Age are mixed. Check other places also. They are not formatted properly. I would also appreciate, if Odd ratios are given at two decimal places both in the table and in the text.

Response: We have re-formatted Table 7 & 8, and odd ratios are given up to two decimal places.

Comments: Table 7A: Var-sufficient preventive measures by govt. Odd ratios for Retired category is too high. Could be due small sample issues. Check it and give foot note on the inappropriate result.
Table 7B: same problem- Odd ratio too high for retired for outcome variable- wear a face mask when going outside.

Response: We provided a reason for these higher OR values “higher OR values were due to small sample size and their responses were similar).

Comments: Table 7A and &B: Several Cells are missing. Give a footnote why there is no OR for those variables.

Response: We provided following foot note “Blank cells reveal that these variables were excluded from logistic regression analysis because these were not significant in chi-squared test.”.

Comments: Table 7B: Clearly define the outcome variables. The interpretations and the definition of outcomes are not consistent. For example, page 20, para 2, "The participants with good knowledge did not allow their children to engage in outdoor activities during COVID-19 (OR 1.751, 95% CI, p<0.05) (Table 7B). According to the table 7B column heading (Presently visit crowded areas, if this is coded as 1), having good knowledge is greater odds to visit crowded areas than those with poor knowledge (reference category). Similarly check other interpretations on page 20 and 21.

Response: We have re-worded the outcomes variables in Table 8. Now the interpretations are consistent with outcomes variables.
